# Characteristics of Crosslinking Polymers Play Major Roles in Improving the Stability and Catalytic Properties of Immobilized *Thermomyces lanuginosus* Lipase

**DOI:** 10.3390/ijms23062917

**Published:** 2022-03-08

**Authors:** Yuhong Mao, Zhenling Cai, Chenxi Zhou, Hangzhen Lan, Xiuyun Ye

**Affiliations:** 1 Fujian Key Laboratory of Marine Enzyme Engineering, College of Biological Science and Technology, Fuzhou University, Fuzhou 350116, China; caizhenling0824@163.com (Z.C.); zcx5621175@163.com (C.Z.); xiuyunye@fzu.edu.cn (X.Y.); 2 State Key Laboratory for Managing Biotic and Chemical Threats to the Quality and Safety of Agro-Products, College of Food and Pharmaceutical Sciences, Ningbo University, Ningbo 315211, China; hangzhen.lan@nbu.edu.cn

**Keywords:** polyethylenimine, chitosan, anti-desorption, hydrolytic activity, esterification

## Abstract

This study aimed to improve the stability and catalytic properties of *Thermomyces lanuginosus* lipase (TLL) adsorbed on a hydrophobic support. At the optimized conditions (pH 5 and 25 °C without any additions), the Sips isotherm model effectively fitted the equilibrium adsorption data, indicating a monolayer and the homogenous distribution of immobilized lipase molecules. To preserve the high specific activity of adsorbed lipase, the immobilized lipase (IL) with a moderate loading amount (approximately 40% surface coverage) was selected. Polyethylenimine (PEI) and chitosan (CS) were successfully applied as bridging units to in situ crosslink the immobilized lipase molecules in IL. At the low polymer concentration (0.5%, *w*/*w*) and with 1 h incubation, insignificant changes in average pore size were detected. Short-chain PEI and CS (MW ≤ 2 kDa) efficiently improved the lipase stability, i.e., the lipase loss decreased from 40% to <2%. Notably, CS performed much better than PEI in maintaining lipase activity. IL crosslinked with CS-2 kDa showed a two- to three-fold higher rate when hydrolyzing p-nitrophenyl butyrate and a two-fold increase in the catalytic efficiency in the esterification of hexanoic acid with butanol. These in situ crosslinking strategies offer good potential for modulating the catalytic properties of TLL for a specific reaction.

## 1. Introduction

*Thermomyces lanuginosus* lipase (TLL) is a versatile biocatalyst, catalyzing various reactions, such as hydrolysis, esterification, transesterification, etc. [1,2]. To be applied at an industrial scale, an immobilized form of TLL is favored to permit its reuse and easy separation from the reaction mixture.

The immobilization of lipases has been explored for decades [1,2,3]. Among various reported approaches, the adsorption of lipases on hydrophobic and porous supports has attracted great attention [3,4,5]. These porous supports not only offer a sufficient surface area by altering their pore size and porosity, but also can be easily recycled. Moreover, lipases adsorbed on these hydrophobic carriers are mostly activated, which is attributed by the so-called “interfacial activation” [3,5]. However, high-temperature organic solvents and substrates/products with detergent properties tend to desorb lipases [3,5]. Consequently, bifunctional agents were introduced to generate intermolecular crosslinking with lipases, thus preventing desorption [3]. The most commonly used one is glutaraldehyde (GA), which requires a sufficient distance between molecules to form intermolecular crosslinking [6].

To improve the intermolecular crosslinking efficiency, functional group-containing polymers are introduced. For instance, polyethyleneimine (PEI), one of the most popular polymers in the design of immobilized biocatalysts [7], comprises amino groups and possesses a high ionic charge density, which can be directly coated on lipases via ionic adsorption [8,9,10]. PEI can form covalent bonds with lipases by adding bifunctional agents as well [11,12]. Another cationic polymer, chitosan (CS), has been used to directly support or entrap lipases and showed promising results in maintaining lipase activity [13]. To apply these polymers in crosslinking enzymes, two concerns should be carefully addressed, i.e., how do their characteristics, such as molecular weight (MW), functional group, etc., affect the coating/crosslinking efficiency and intensity? Additionally, would a different coating/crosslinking intensity affect enzyme performance? Using large polymers was suggested in a previous work [8], given that a polymer with a large MW offered the possibility to crosslink several enzyme molecules, especially for those far with each other. However, the tethering of polymers with a large MW, even larger than the enzyme itself, might increase the risk of covering the substrate tunnel or catalytic cavity of the enzyme. A similar effect happens with densely crosslinked polymers. Therefore, a compromise should be made between efficient intermolecular crosslinking to avoid enzyme leakage and a less dense crosslinking to ensure the enzymatic activity, especially toward those substrates with large MWs. Compared with PEI, CS possesses much less charge intensity and comprises both hydroxyl and amino groups. The crosslinking of GA with hydroxyl groups must be conducted in acidic conditions, while amines prefer to crosslink with GA in neutral and basic conditions [14]. Therefore, a much less dense crosslinking of GA with CS than that with PEI can be expected when the reaction is conducted at a basic condition. Except for different crosslinking densities, the microenvironment around immobilized lipases might vary significantly due to the different choices regarding to use PEI or CS as the crosslinking polymer. Several works [15,16] inferred that the alteration of the microenvironment around lipases significantly contributed to their distinctive selectivity toward substrates with different properties, such as chain length. Therefore, it is necessary to check the substrate selectivity of immobilized lipase after its crosslinking with different polymers.

Prior to crosslinking, the distribution of adsorbed lipase molecules through the porous support requires a modulation, because the crosslinked products might differ greatly when immobilized lipase molecules distribute heterogeneously or homogenously. For instance, if the intra-particle diffusion of lipases is much slower than its incorporation on the pore entrance, lipase molecules might be closely packed there [17]. Such a heterogeneous distribution may prevent the diffusion of polymers, especially those with large MWs, and the dense crosslinked products may form on the external part of the pores, even leading to a pore blockage. Conversely, a monolayer and homogenous distribution of immobilized lipase molecules not only minimizes the adverse impact on the diffusion of polymers, but also contributes to an increase in the specific activity of immobilized lipase.

This study aimed to improve the stability and catalytic performance of TLL adsorbed on a hydrophobic and porous support. Firstly, the influence of certain parameters on the adsorption efficiency (enzyme loading amount, activity, and specific activity) was investigated using a DOE design. An isotherm analysis was performed as well to elucidate the adsorption process. Secondly, an in situ crosslinking of immobilized lipase molecules with PEI or CS was conducted to further improve the performance. In addition, this study focused on clarifying the influence of polymers’ (PEI and CS) characteristics on the crosslinking efficiency and the catalytic properties of TLL.

## 2. Results and Discussion

### 2.1. Adsorption Process

#### 2.1.1. Influence of Different Factors on the Adsorption Process

Details on the DOE analysis are described in Table 1. The ΔG value was calculated to evaluate the nature of the adsorption process. A negative ΔG value raging from −20 to 0 kJ/mol corresponds to spontaneous physisorption, and a higher absolute value indicates a more energetically favorable process [18]. According to the ΔG values summarized in Table 1, the immobilization of TLL on the support occurred via physisorption. The absolute value of ΔG here, ranging from 0 to 5.42 kJ/mol, was generally smaller than the ones reported by Alves et al. [18]. A support with higher hydrophobicity in that study than the one here (water contact angle around 97 ± 2°) was probably applied.

Factors that significantly affect the loading protein amount, hydrolytic activity, and specific (hydrolytic) activity are summarized in Table S1, and their individual effects are described in Figures S1–S3 (in the Supplementary Materials). As shown in Table S1 and Figure S1A, increasing the initial lipase concentration from 0.1 to 2 mg/mL significantly increased the loading protein amount. Accordingly, activity increased greatly at the beginning, while a slight decrease in activity at high initial concentrations (more than 1 mg/mL) was observed, which led to a minor decline in the specific activity (Figure S3C). Enzyme crowding at high initial concentrations might account for a such decline [19], which should not happen in this case. The support (70% water content) has a BET surface area of around 130 m^2^/g (dry), and TLL has a molar mass of ~30 kDa and a hydration radius about 5.3 nm [18]. Assuming that a monolayer of lipase molecules adsorbed on the support, then only around 80% of the support surface was covered by lipase molecules even at the highest loading protein amount (54.30 mg/g wet support). Therefore, either there were some pores that were too hard for lipase molecules to diffuse in (due to the pore size or the support hydrophobicity [20]), or the firstly adsorbed lipase molecules prevented other molecules from entering pores (due to electrostatic repulsion or protein–protein interaction [19]).

Pronounced increases in loading protein amount, activity, and specific activity were determined at pH 5, where TLL was generally not charged, as it was close to its isoelectric point (pH 4.6) [21]. The ionic strength (NaCl) did not significantly affect the loading protein amount, while it led to a decline in activity (Figure S2C). This was probably due to a faster adsorption at the beginning when high concentrations of NaCl were added (data not shown), leading to more severe enzyme crowding. In addition, a significant decrease in loading protein amount with increasing ethanol addition was observed (Figure S1B). This effect was more pronounced when a high initial lipase concentration was applied (Figure S1D). The addition of ethanol is reported to improve the immobilization yield of lipase on hydrophobic supports, as it might improve the access of lipase into the pores with its hydrophobic surface [20]. An opposite observation in this study was probably due to the different natures of the supports applied (ours seemed to be less hydrophobic). Temperatures from 10 to 40 °C did not significantly affect the loading protein amount, while a low temperature improved the specific activity of lipase at unsuitable pH values (pH 3 and 8).

The immobilization yields of both protein and activity were calculated in Table 1. At a low initial amount of protein, the immobilization yield of protein reached 100%, and even at the highest initial protein amount in this study, a 90.50% immobilization yield of protein was achieved. The immobilization yield of protein highly depends on the immobilization conditions. Compared with the activity of free lipase, the yield of activity ranged from 0 to 79.46%, indicating a loss in hydrolytic activity after immobilization, at least to the olive oil substrate. This was probably due to the diffusional limitation in the substrate to the immobilized lipase molecules located inside the pores. However, the dramatically decreasing activity at pH 3 with the addition of ethanol and/or NaCl was probably due to the inactivation of lipase.

#### 2.1.2. Adsorption Kinetic and Isotherm Analysis

**Figure 1:**
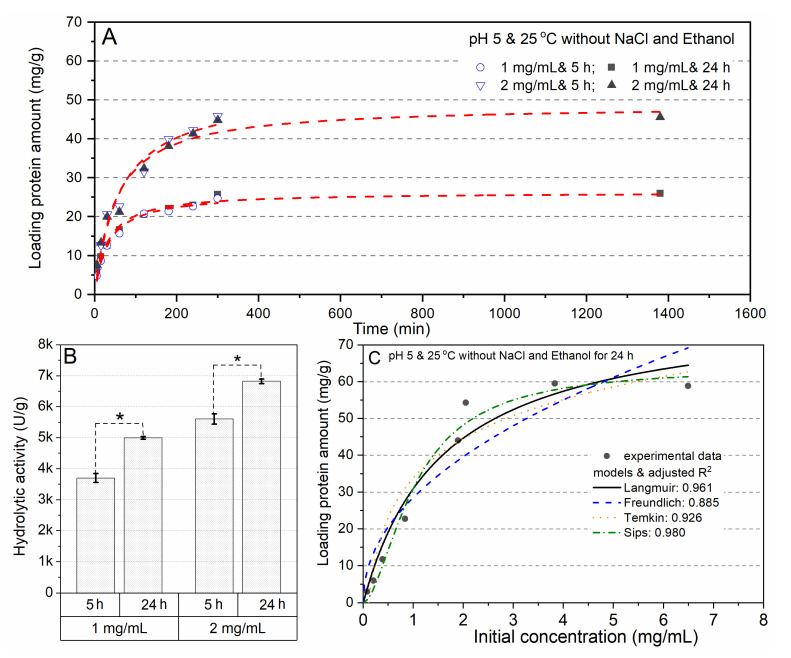
The adsorption kinetic of lipase at an initial protein concentration of 1 mg/mL or 2 mg/mL (**A**); the hydrolytic activity of immobilized lipases with different incubation times (**B**); non-linear isotherm models for the equilibrium data of lipase adsorption (**C**). * Indicates a significant difference (* *p* < 0.05).

In accordance with the influence of investigated parameters on the physisorption of lipase, the adsorption kinetic and isotherm were evaluated at pH 5 and 25 °C without any additions. As described in Figure 1A, the adsorption process achieved the saturation after 5 h, while the hydrolytic activity was significantly improved by extending the incubation time to 24 h (Figure 1B). The Sips isotherm model consists of a combination between Langmuir and Freundlich, effectively fitted to the experimental data with the highest adjusted R^2^ value. This model reflects a monolayer sorption capacity characteristic even at a high initial protein concentration, and also assumes that this monolayer adsorption takes place homogenously [18].

### 2.2. Efficiency of Various Modifications

To ensure the high specific activity of the adsorbed lipase, the biocatalyst (named as IL) with a moderate loading amount (initial lipase concentration of 1 mg/mL) was developed at the optimized conditions (pH 5 and 25 °C without any additions). IL had a protein loading amount of 24.75 ± 2.12 mg protein per gram of wet support, corresponding to an immobilization yield (protein) of 89.17 ± 7.07% and around 40% surface coverage, and its initial hydrolytic activity toward olive oil was 6213.72 ± 102.60 U/g (based on wet support). All the modifications are summarized in Table 2.

#### 2.2.1. The Efficiency of Physical Coating

**Figure 2:**
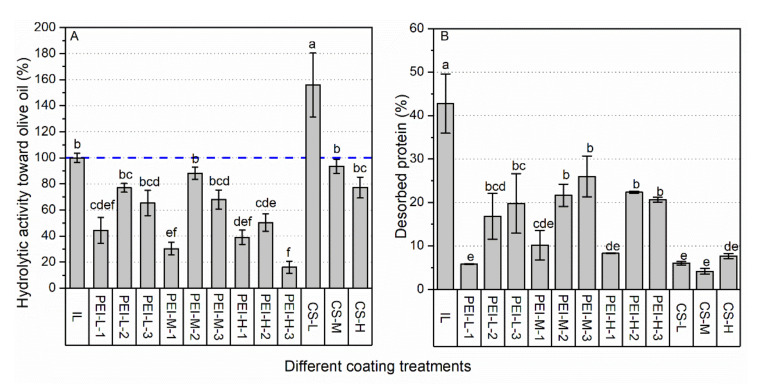
The effects of physical coating on hydrolytic activity (**A**) and stability against desorption (**B**). Different lowercase letters indicate significant differences among different coating treatments (*p* < 0.05).

As shown in Figure 2A,B, 42% of the lipase molecules was desorbed from IL, which was greatly reduced after the physical coating of PEI or CS was applied. Although a better protection was observed when a high concentration of PEI (30%) was applied, it led to more than 60% decrease in hydrolytic activity. Moreover, extending the incubation time from 1 to 24 h was not beneficial to the anti-desorption efficiency, and led to a decrease in activity, especially for the preparations using PEI-H (75 kDa). PEI with 2 kDa, which was never investigated in previous works, showed roughly better protection than PEI with 25 or 75 kDa here. This was probably attributed by the strong diffusional capacity of PEI with such a low MW, meaning that a better coating coverage can be expected. Differing in the crosslinking of several enzyme molecules to PEI with a large MW, 2 kDa PEI could enhance the interaction affinity between the lipase molecules and the support, and minimized the risk of covering the active center of the lipase.

According to the protective effects of PEI at different conditions, the concentration of 0.5% and 1 h incubation was applied for the modifications with CS. Even CS-M, not to mention CS-H, had a MW in the 50–190 kDa range, which was much larger than the applied PEI. Thus, a less effective protection of CS-M and CS-H than PEI was established initially because of their insufficient diffusion. Surprisingly, CS with all three MWs showed much better anti-desorption effects than PEI under the same incubation conditions. Furthermore, the preparations coated by CS-L had around a 50% increase in its activity compared with IL. With the increasing MW, the activity decreased accordingly, while none of them showed significantly decreased activity compared with IL. These results indicated that the interaction between CS and lipase, such as the formation of hydrogen bonds, improved the hydrolytic activity. Similarly, a recent study [22] found that the CS-involved CLEAs of *Aspergillus niger* type A feruloyl esterase displayed much higher activity than its free form, and they speculated that CS provided a catalytic microenvironment which could effectively concentrate substrates with long alkyl chains.

#### 2.2.2. The Efficiency of Chemical Crosslinking

Both PEI and CS were proven to protect lipase from desorption even with a simple physical coating. Such encouraging results roused our interest in exploring whether it was possible to further improve the stability with the in situ generation of more compactly crosslinked polymers involving lipase molecules. To achieve such crosslinking, GA was applied as the bifunctional agent. According to the pretest (see Figure S4 in the Supplementary Materials), the GA concentration of 1% and 15 min incubation at pH 7.2 and 25 °C was chosen. To confirm whether an efficient diffusion of PEI/CS would affect the crosslinking efficiency, a pre-incubation with PEI or CS for 45 min before adding GA was conducted and compared with the simultaneous addition of the polymer and GA.

**Figure 3:**
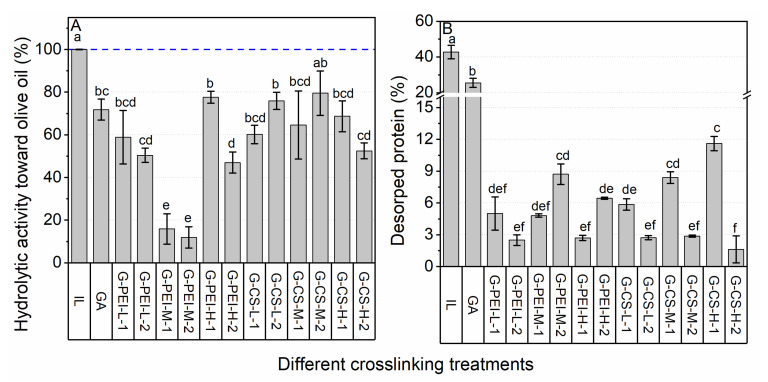
The effects of chemical crosslinking on hydrolytic activity (**A**) and stability against desorption (**B**). Different lowercase letters indicate significant differences among different crosslinking treatments (*p* < 0.05).

**Figure 4:**
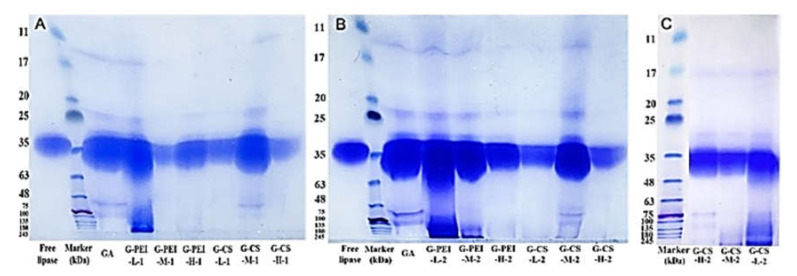
The crosslinked products analyzed via SDS-PAGE were described in (**A**–**C**). Different letters indicate statistically significant differences. Certain lanes were deleted from G due to the experimental mistakes in sample loading. Its original SDS-PAGE is shown in the Supplementary Materials (Figure S5).

Compared with PEI, CS performed much better in maintaining lipase activity (Figure 3A). The GA-rendered crosslinking of lipase without polymers not only led to a 20–30% decrease in activity, but also offered insufficient protection (Figure 3). This was due to the fact that GA was too small to form effective intermolecular crosslinks for lipase molecules which were located relatively far away from each other, evidenced by the fact that most lipase molecules remained as monomers (Figure 4A,B). With the addition of polymers, all these crosslinked preparations had a desorption amount less than 12%. The pre-incubation with CS significantly improved its anti-desorption, especially for CS-M and H. This was probably due to their relatively slow diffusion. Conversely, pre-incubation did not improve the anti-desorption effect of PEI, which might have a relatively fast diffusional capacity thanks to its intense positive charge (lipase was negatively charged in a pH 7.2 incubation buffer) and relatively small MW. This fast diffusion was proven by the obviously observed intermolecular crosslinking in the preparations using PEI (Figure 4A,B), particularly for PEI-L. Therefore, PEI-L was proven to act as a bridging unit that could form sufficient intermolecular crosslinks among lipase molecules that located relatively far from each other. Moreover, it was believed that intermolecular crosslinks were formed as well when PEI-H was applied, despite the fact that it was not directly confirmed by SDS-PAGE. This was probably due to the huge MW of its crosslinked products whereby, as for PEI-M, the MW of the crosslinked products was already above 245 kDa. The lowest hydrolytic activity was observed for the preparations with PEI-M. Furthermore, pre-incubation caused a significant decrease in hydrolytic activity for the preparations crosslinked with PEI-H, suggesting that intense crosslinking with PEI might cover the active center of lipases, and thus impaired the activity.

For CS, the intermolecular crosslinking was only observed for the ones using CS-M, while its SDS-PAGE pattern was quite similar to the preparation using GA along. For preparations using CS-L or CS-H, although no clear bonds for intermolecular crosslinking were visible, their monomer bonds decreased significantly (Figure 4A,B). Therefore, it was difficult to confirm whether CS was involved as a bridging unit to form intermolecular crosslinking of lipase molecules, or if it just underwent a self-polymerization to form a mesh protecting the lipase leakage. Therefore, we extended the boiling time (during the rupture) from 10 to 30 min for the preparations crosslinked with CS. Figure 4C suggested that intermolecular crosslinking among lipase molecules was formed when CS-L or CS-M was applied, while for CS-H, no evidence was able to confirm whether successful intermolecular crosslinking occurred between itself and the lipase molecules.

### 2.3. Characterization of Immobilized Lipase with Various Modifications

**Figure 5:**
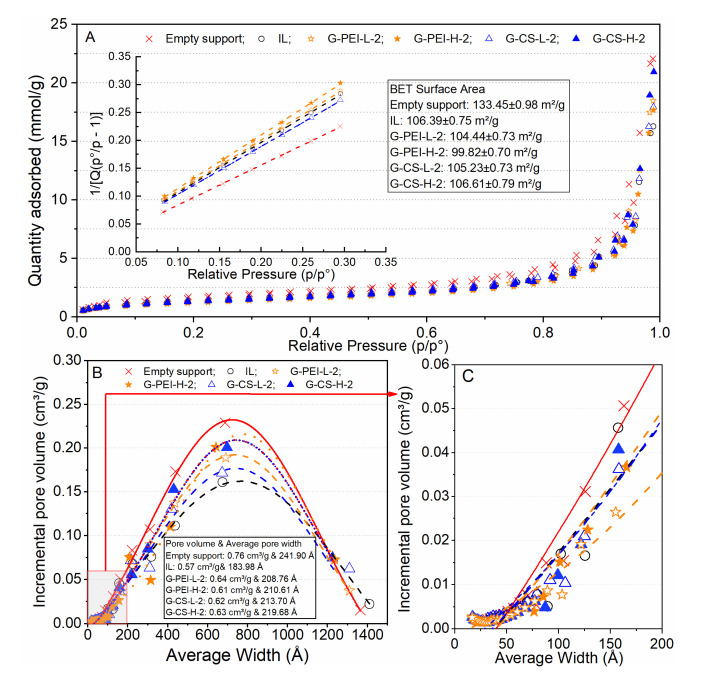
BET full nitrogen adsorption isotherm (**A**), where the BET plot is shown in the inset; pore size distribution curve (**B**); graph showing that the distribution of pore sizes smaller than 200 Å was enlarged (**C**).

**Figure 6:**
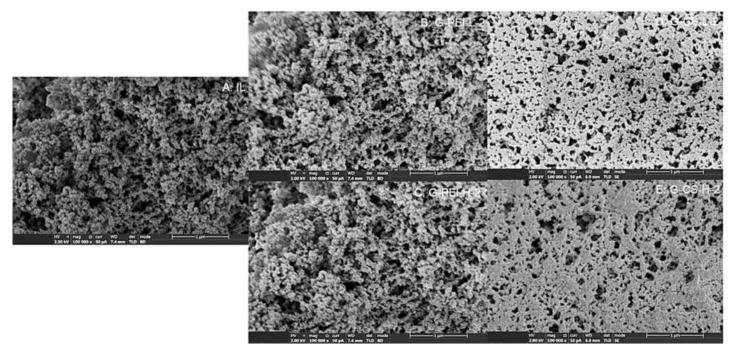
SEM micrographs of immobilized lipase with different modifications. Physical adsorption (**A**); chemically crosslinking with PEI-L (**B**) and PEI-H (**C**); chemically crosslinking with CS-L (**D**) and CS-H (**E**).

As shown in Figure 5A, the empty support had a BET surface area of around 133 m^2^/g (dry). With the loading amount of 24.75 ± 2.12 mg protein per gram of wet support (82.50 ± 7.07 mg/g dry support), the BET surface area decreased by 20%. The BET surface area declined insignificantly after the crosslinking with PEI or CS, although a decrease of around 6.6% was observed when PEI-H was applied. Interestingly, the crosslinking with polymers contributed to a 7–15% increase in the pore volume and a 12–20% increase in the average pore size. These results correlate well to the corresponding SEM micrographs. As shown in Figure 6, a porous structure was clearly observed before and after the crosslinking with PEI or CS. The insignificant increase in the average pore size was probably due to the re-arrangement of the lipase molecules in the support, which might happen during the additional incubation period (1 h for crosslinking and 18 h for reduction). This rearrangement was probably a result of the interaction between lipase molecules and the applied polymer, or even just because of the formed hydrogen bonds with water molecules. Such re-arrangement was observed in a pioneering work [23], where adsorbed lipase was distributed throughout the porous support by increasing the incubation time. The addition of PEI or CS led to a slight decline in the measured maximal pore size, while the volume percentage of the major pore size (600–800 Å) increased by large amount, especially for preparations using PEI or CS with a large MW. Thus, it is speculated that both PEI and CS with a large MW preferably diffused into large pores. Correlating to their anti-desorption effectiveness (3.2), the 42% desorbed lipase from IL could primarily be located in these large pores. For those pores with a size smaller than 200 Å, insignificant changes were observed, except for PEI-L, with which the volume percentage of small pores declined slightly. This change confirmed the strong diffusional capacity of PEI-L.

### 2.4. Hydrolytic Activity and Esterification Capacity

#### 2.4.1. Hydrolytic Activity towards Different Substrates

TLL is a typical lipase requiring interfacial activation, as its active center is covered by a lid composed of hydrophobic residues, which renders TLL more capable of hydrolyzing long-chain substrates [21]. p-Nitrophenyl butyrate (NPB) is a water-soluble short-chain substrate for esterase, and it is widely used in many works [24,25,26,27] to monitor the hydrolytic activity of lipases. p-Nitrophenyl palmitate (NPP) is a long-chain substrate for lipase, which is commonly applied in an emulsion or is dissolved in organic solvents, such as heptane [28].

**Figure 7:**
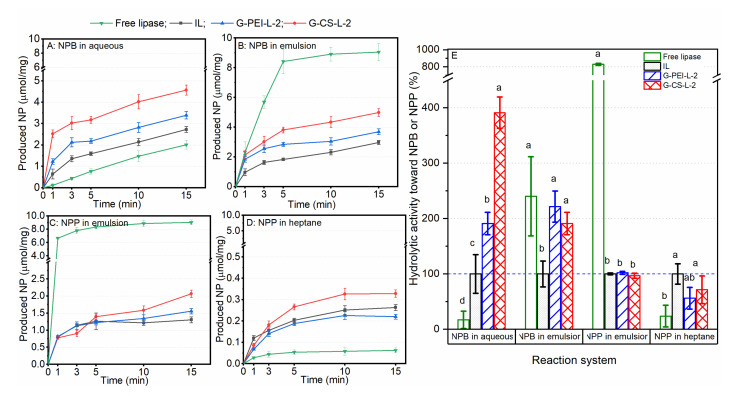
The hydrolysis of p-nitrophenyl butyrate (NPB) in an aqueous solution (**A**) and in an emulsion (**B**); the hydrolysis of p-nitrophenyl palmitate (NPP) in an emulsion (**C**) and in heptane (**D**). The initial hydrolytic reaction rate (within the first 1 min) was calculated as hydrolytic activity and the activity of IL was taken as 100% (**E**). The hydrolytic capacity was described by the amount of NP produced per milligram of dried immobilized lipase preparations or dried lipase powder (free form). Different lowercase letters in (**E**) indicate significant differences among the applied biocatalysts for each reaction system (*p* < 0.05).

As shown in Figure 7A, all biocatalysts were able to hydrolyze NPB, while the activity of free lipase in aqueous medium was much lower compared to the immobilized ones. This was due to the lack of interfacial activation for free lipase, which was proven by its largely increased hydrolytic rate in the emulsion medium (Figure 7B). Conversely, the lipase immobilized on a hydrophobic support was already in its active form; thus, a considerable hydrolytic activity in both aqueous and emulsion was observed. Moreover, the modifications with PEI or CS significantly improved the hydrolytic activity of IL toward NPB. All the biocatalysts exhibited considerable activity toward emulsified substrate NPP, although the free lipase showed an eight-fold higher activity compared to the immobilized lipases (Figure 7C,E). When NPP was dissolved in pure heptane, the detectable activity for all biocatalysts decreased substantially, especially for free lipase (Figure 7D). Due to the high hydrophobicity of heptane, free lipase (dissolved in buffer) was not able to be dispersed homogeneously; rather, it agglomerated as one small droplet, even when a 1000 rpm shaking speed was applied. Although immobilized lipase preparations were well dispersed, it was too difficult for the substrate in heptane to diffuse into pores, as an aqueous buffer was filled in each pore (a water molecule was necessary for the hydrolytic reaction). Therefore, severe diffusional limitations could account for such a low reaction rate for both free and immobilized lipases. In addition, free lipase showed higher hydrolytic activity toward NPP than NPB in the same emulsion medium. This corresponds well to its natural catalytic property, i.e., TLL displays a high affinity for long-chain substrates [29]. However, such preference disappeared after its immobilization, as IL showed similar, even higher, hydrolytic activity toward NPB than NPP. Once an enzyme was immobilized, its mobility declined significantly. Accordingly, the affinity of this enzyme to substrates, to some extent, turns from active selection to passive acceptance. Therefore, the faster diffusion of NPB in a porous support filled with an aqueous buffer could explain the increased activity of immobilized lipases.

#### 2.4.2. Esterification Capacity

To check the substrate selectivity of various immobilized lipases in esterification, hexanoic acid and oleic acid were reacted with ethanol or butanol. Esterification catalyzed by a lipase is a reversible reaction; therefore, the produced amount of ester was monitored at 0.5 and 2 h.

**Figure 8:**
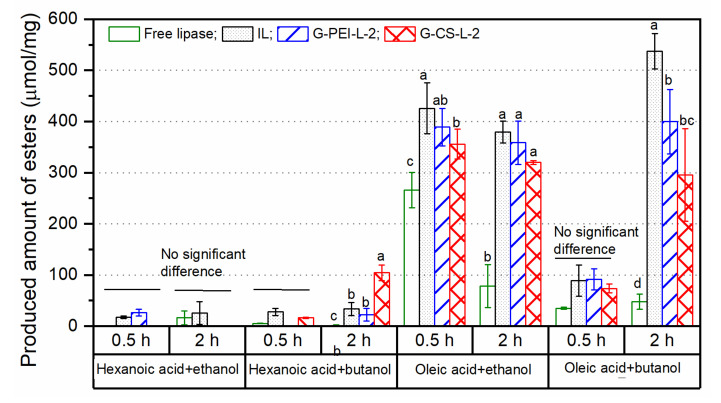
Esterification capacity toward different pairs of fatty acid and alcohol. The esterification capacity was described by the amount of ester produced by per milligram of dried immobilized lipase preparations or dried lipase powder (free form). Different lowercase letters indicate significant differences among the applied biocatalysts for each reaction at 0.5 or 2 h (*p* < 0.05).

As shown in Figure 8, the chain length of fatty acids played an important role in TLL-catalyzed esterification. All lipase preparations showed low activity toward hexanoic acid, particularly in these reactions with ethanol. Likewise, Kovalenko et al. [30] reported the low esterification rate when catalyzed by TLL for fatty acids with less than seven carbons. Notably, G-CS-L-2 exhibited a two-fold capacity increase in the esterification of hexanoic acid with butanol compared with IL. Toward oleic acid, all immobilized lipases exhibited much higher activity than the free one. Compared with butanol, the initial esterification rate between oleic acid and ethanol was much faster for all lipase preparations. Noro et al. [31] found that native free TLL showed higher affinity toward alcohols with three or less carbons than those with a longer chain length during the esterification of oleic acid with various alcohols. However, the reverse reaction happened simultaneously during the esterification between oleic acid and ethanol, as an even lower amount of ester was detected at 2 h than that at 0.5 h, especially for free lipase. This was probably due to the overly fast accumulation of the by-product, i.e., water molecules, which could not be removed by the molecular sieve efficiently. Such a reverse reaction was significantly reduced in the esterification of oleic acid with butanol catalyzed by immobilized lipase preparations. This could be caused by the hydrophobic nature of the applied support, which might repel the slowly increased water molecule from the immobilized lipase. However, the modified samples exhibited decreasing esterification capacity at 2 h, compared with IL. An aqua microenvironment around the immobilized lipase was probably created along with the reaction, leading to a reverse reaction.

### 2.5. Stability and Reusability Analysis

**Figure 9:**
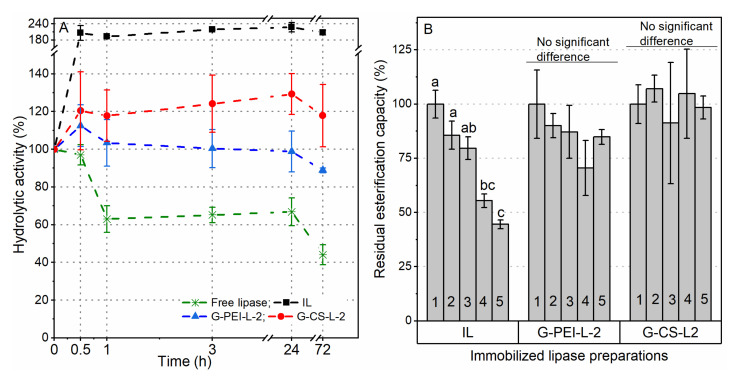
Stability of free and immobilized lipase preparations at 50 °C with the incubation period up to 72 h (**A**); the reusability of immobilized lipase preparations (**B**). Different lowercase letters indicate significant differences among different reaction cycles for each biocatalyst (*p* < 0.05).

As shown in Figure 9, a 40% decrease after the first hour of incubation at 50 °C was detected for free lipase, while its activity remained constant up to 24 h and then declined to 40% of its initial activity. TLL is a thermal stable lipase and has been reported to maintain its activity at up to 55 °C, while it is one of the lipases highly tending to form dimers [21]. Speculatively, the decreased activity in the first hour was probably due to the formation of dimers, rather than the thermal induced denaturation. On the contrary, the activity of immobilized lipase preparations increased greatly after the first 0.5 h incubation period, where a double activity was detected for IL. Indeed, all biocatalysts used here were vacuum-dried completely, while water molecules are necessary for the hydrolytic reactions. Therefore, the dry sample required a wetting course during the activity measurement, and the modifications with PEI or CS seemed to accelerate this wetting course. Although no significant difference in activity for immobilized lipase preparations was detected up to 72 h, a slight declining trend appeared after 24 h, indicating that long-term thermal treatment did insert stress on the lipase structure, and thus on its activity. Regarding the reusability, no significant difference was detected in the esterification capacity during consecutive applications of G-PEI-L-2 and G-CS-L-2, while for IL, a clear decrease was observed after three cycles (Figure 9). As all immobilized lipase preparations showed high thermal stability (Figure 9), such a decrease for IL was probably due to the desorption of lipase molecules from the support.

## 3. Materials and Methods

### 3.1. Materials

Lipase from *Thermocycles lanuginosus* (powder, 3.5% protein content) was acquired from Fuda Biotech Co., Ltd. (Ningde, China) Methylmethacrylate–butylmethacrylate–divinylbenzene–ethylvinylbenzene copolymer resin (support) and polyethyleneimine (PEI) (MW 2, 25, 75 kDa) were kindly donated by Lanxess AG (Cologne, Germany) and BASF Shanghai Coatings Co., Ltd. (Shanghai, China), respectively.

Arabic gum, p-nitrophenyl butyrate (NPB), p-nitrophenyl palmitate (NPP), p-nitrophenol (NP), chitooligosaccharides chitosan (CS-L), chitosan with MW 50–190 kDa (CS-M), chitosan with a viscosity of 200–800 cp (CS-H), glutaraldehyde (GA), sodium cyanoborohydride, ethanolamine, and 4-morpholinethanesulfonic acid-2-(N-morpholinyl)-ethanesulfonic acid (MES) were purchased from Sigma-Aldrich. Olive oil (low acidity), oleic acid, hexanoic acid, ethanol, butanol, and other chemical reagents (all of analytical grade) were purchased from Aladdin (Shanghai, China) Co., Ltd. or Sinopharm Group Co., Ltd. (Shanghai, China).

### 3.2. Adsorption of Lipase

Free lipase was immobilized on the support via adsorption. The wet support was firstly soaked in methanol (1:10, *w*/*v*) for 30 min, and then methanol was removed via vacuum filtration and the support was washed by 100 mL of ultra-pure water 3 times at room temperature to completely remove contaminants in its original package. The support was dried by vacuum filtration for 10 min before each immobilization experiment. Lipase powder was suspended in ultra-pure water with magnetic stirring at 100 rpm for 1 h at 25 °C. The suspension was centrifuged at 6000 g for 10 min to remove its insoluble additives, and the collected supernatant was mixed with different buffers according to factorial screening design using DOE. One gram of wet support was soaked in 30 mL lipase solutions and shaken at 200 rpm in a shaking incubator (ZWYR-240, Labwit Scientific) with a constant temperature for 24 h. Then, 200 µL of the supernatants was withdrawn at various time intervals. After immobilization, each sample was collected by vacuum filtration and washed with 100 mL of ultra-pure water, then stored at 4 °C after vacuum filtration. For long-term storage, samples were firstly vacuum dried at 35 °C for 24 h and then stored at 4 °C.

The effects of pH, temperature, initial lipase concentration, ionic strength (NaCl addition), and ethanol addition on this adsorption process were evaluated using the DOE approach. At the optimized condition, an increasing initial lipase concentration from 0.1 to 6 mg/mL was applied to investigate the adsorption isotherm.

Protein loading amount per gram of wet support (*q_e_*, mg/g) was calculated according to Equation (1). The protein concentration was determined using a BCA protein assay kit according to the manufacturer’s instructions (KeyGEN Biotech, Nanjing, China).

(1) $$q_{e}=\frac{(C_{o}-C_{t})\times V}{m}$$

where *C_o_* and *C_t_* (mg/mL) are the protein concentrations of lipase solution at the beginning and at time *t*, respectively. *V* (mL) is the total volume of lipase solution used for immobilization and *m* (g) is the weight of empty support (wet).

Immobilization protein yield (η_protein_, %) was calculated according to Equation (2).

(2) $$\eta_{\mathrm{protein}}\left(\%\right)=\frac{q_{e}}{C_{o}\times v_{o}}$$

where *q_e_* is the final protein loading amount per gram of wet support (mg/g), *C_o_* is the initial protein concentration (mg/mL), and *V_o_* is the initial volume of protein solution (mL), which here is 30 mL.

The Gibbs free energy (Δ*G*) for adsorption process was calculated according to Equation (3) [18].

(3) $$\Delta G=-RT\ln\frac{C_{0}}{C_{t}}$$

where *R* is the gas universal constant (8.314 × 10^−3^ kJ/mol K) and T is the temperature (298.15 K), while *C_o_* and *C_t_* (mg/mL) are the initial protein concentration and residual protein concentration at equilibrium, respectively.

Experimental data on the adsorption isotherm were fitted to the isotherm models of Langmuir (Equation (4)), Freundlich (Equation (5)), Temkin (Equation (6)), and Sips (Equation (7)) [18].

(4) $$q_{e}=\frac{q_{max}\times C_{0}}{K_{l}}$$

(5) $$q_{e}=K_{f}\times c_{0}^{\frac{1}{n}}$$

(6) $$q_{e}=\frac{RT}{K_{t}}\times \ln\left(A_{t}\times c_{0}\right)$$

(7) $$q_{e}=\frac{K_{s}\times c_{0}^{\beta }}{1+\alpha \times c_{0}^{\beta }}$$

where *q_e_* and *C_o_* are the adsorption capacity (loading protein amount, mg protein/g wet support) and initial protein concentration (mg/mL), respectively. *K_l_* is the Langmuir constant related to the energy of adsorption (mL/mg protein), and *q_max_* is the maximum adsorption capacity (mg protein/g wet support). *K_f_* is the Freundlich isotherm constant (mL/mg support), and *n* is the Freundlich exponent (dimensionless). *K_t_* is the Temkin isotherm constant (J/mol), *R* is the gas universal constant, *T* is the experimental temperature (298.15 k), and At is the Temkin isotherm equilibrium binding constant (mL/mg support). *K_s_* is the Sips isotherm model constant (mL/mg), and α and β are Sips model constant (mL/mg) and exponent (dimensionless), respectively.

### 3.3. Modifications of Physically Adsorbed Lipase

To achieve a physical coating, 100 mg of wet immobilized lipase (IL) samples were suspended in 19 mL phosphate buffer (pH 7.2, 50 mM), and 1 mL of CS solution or PEI solution was added. The samples were gently shaken (150 rpm) for different periods at 25 °C. For chemical crosslinking modification, 100 mg of wet IL samples in 18 mL phosphate buffer (pH 7.2, 50 mM) were firstly coated by 1 mL CS or PEI for 45 min, and then chemically crosslinked by adding 1 mL of 1% GA solution for 15 min; alternatively, the IL samples were directly crosslinked by GA for 15 min, with or without the addition of CS or PEI simultaneously. The GA-crosslinked samples were further reduced with sodium cyanoborohydride and ethanolamine at pH 5.5 and 25 °C for another 20 h. Modified IL samples were washed with 100 mL of ultra-pure water and vacuum filtered, then were stored at 4 °C or vacuum-dried at 35 °C overnight (then stored at 4 °C).

### 3.4. Lipase Catalyzed Reactions

#### 3.4.1. Hydrolytic Reactions

Hydrolytic activity was determined by measuring the free fatty acid released during the hydrolysis of olive oil by free or immobilized lipase in an emulsion [32]. One unit (U) of hydrolytic activity was defined as the amount of enzyme preparation that hydrolyzes olive oil to release 1 μmol free fatty acids per minute at experimental conditions. Specific activity (SA, U/mg protein) for immobilized and free lipase were calculated according to Equations (8) and (9), respectively. The immobilization activity yield (η_activity_, %) was calculated using Equation (10).

(8) $${\mathrm{SA}}_{immobi}=\frac{A_{immobi}}{q_{e}}$$

(9) $${\mathrm{SA}}_{free}=\frac{A_{free}}{m}$$

(10) $$\eta_{\mathrm{activity}}\left(\%\right)=\frac{{\mathrm{SA}}_{immobi}}{{\mathrm{SA}}_{free}}$$

where *A_immobi_* (U/g) and *A_free_* (U/g) are the hydrolytic activity per gram of immobilized lipase preparation and lipase powder (free form), respectively. *q_e_* (mg/g) and m (mg/g) are the protein amount (mg) per gram of immobilized lipase preparation and lipase powder (free form), respectively.

Lipase activity toward substrate NPB or NPP was determined spectrometrically at 410 nm according to the standard curve using the absorbance of standard NP solution in phosphate buffer (50 mM, pH7.5). The dried immobilized lipase preparations were soaked in the same phosphate buffer for 30 min at 25 °C to be wetting before each measurement, and the activity was described by the amount of NP produced by per milligram of dried biocatalysts. Free lipase was firstly dissolved in phosphate buffer (50 mM, pH 7.5) at the concentration of 1 mg/mL (protein), and then 10 μL was applied to each reaction, the activity was calculated for per milligram of dried lipase powder (3.5% protein content).

Then, 1 mL of 30 mM NPB in phosphate buffer (50 mM, pH7.5) was hydrolyzed at 40 °C and 200 rpm, during which 20 µL of each sample was taken out at intervals of 1, 3, 5, 10, and 15 min and diluted in 1 mL phosphate buffer (50 mM, pH7.5). The reaction medium for NPP was prepared according to the method described by Šibalic et al. [33] with modifications. Specifically, 30 mM NPP was prepared in pure heptane or a heptane–phosphate buffer (1:1, *v*/*v*) emulsion. To form a stable emulsion, phosphate buffer (pH 7.5, 50 mM) contained 0.3% (*w*/*w*) gum arabic, and the mixture was mixed using a vortex for 30 s, followed by ultrasonic mixing for 10 min in ice (80% power, 20 KHz, 3 s on/6 s off). Then, 40 µL of each sample during the reaction (40 °C and 200 rpm) was drawn at different time intervals and mixed with 1 mL phosphate buffer. When a clear separation between the aqueous and heptane solutions in diluted samples was observed (around 5 min), 300 µL of samples was taken carefully from bottom aqueous solutions to a 96-well plate and measure at 410 nm. This emulsion medium was used to prepare the NPB substrate as well to check the hydrolytic activity of lipase toward the same substrate in different solvents.

#### 3.4.2. Esterification Reactions

Esterification reactions were carried out using 0.6 M of different pairs of fatty acid and alcohol (molar ratio of 1:1) in heptane (20 mL total reaction volume). Here, 10–15 mg of free lipase powder (protein content 3.5%) or immobilized lipase preparations (dry) were used to catalyze the reactions for 0.5 or 2 h at 50 °C and 200 rpm (air bath). Prior to reaction, the biocatalysts were vacuum-dried at 35 °C overnight, and the reagents and heptane were dried overnight using molecular sieves (pore size 3 Å). Then, 10% (*w*/*w*) molecular sieves were added to the reaction medium during each esterification reaction. The esterification capacity was evaluated using the converted amount of free fatty acid (µmol) per milligram of the applied biocatalyst (dried immobilized lipase preparation or free lipase powder), which was determined using titration as described by Kiran et al. [34].

### 3.5. SDS-PAGE Analysis of Chemically Crosslinked Samples

SDS-PAGE analysis was performed in 15% resolving gel with 5% stacking gel. Here, 10 mg of each immobilized lipase preparation (dry) was suspended in 200 µL of rupture buffer (8 M urea, 6% SDS, and 10% mercaptoethanol, *w*/*w*) and incubated in boiling water for 10 min. Then, 20 µL of each ruptured sample was mixed with 5 µL loading buffer and boiled again for 5 min. After centrifugation at 13,000 rpm for 3 min, 20 µL of supernatants was loaded. The samples were run in an ice bath at 80 V for stacking gel and at 110 V for resolving gel. A broad range marker from 5 to 245 kDa was applied.

### 3.6. Characterization of Immobilized Lipase Preparations

The specific surface areas of dried samples were determined using the nitrogen adsorption–desorption method (BET, Brunauer–Emmett–Teller, ASAP 2460, Micromeritics Instrument Corporation, Norcross, GA, USA). The pore size distribution and pore volume were obtained using BJH (Barret–Joynerand–Halenda) desorption isotherms. Selected samples were imaged using the Thermo Scientific™ (Waltham, WA, USA) Verios G4 scanning electron microscope (SEM). Samples were dried and sputter-coated with gold prior to SEM imaging. SEM images were acquired at 10 kV accelerating voltage with a magnification of 100,000×.

### 3.7. Stability and Reusability Evaluation

The stability against detergent (1% triton × −100) desorption at 50 °C was evaluated to assess the effectiveness of various modifications. Here, 50 mg immobilized lipase preparation (wet) was incubated in 1 mL 1% triton × −100 solution (*w*/*w*, in 50 mM phosphate buffer, pH 7.5) at 50 °C. Then, 80 µL of supernatant was withdrawn at 30 min, 1, 2, and 3 h and the protein content in each was quantified using the BCA method. The desorbed protein was described as the percentage of the initial adsorbed protein (final loading protein amount).

The stability of the selected samples against thermal stress was evaluated by determining their residual hydrolytic activity toward olive oil during the incubation in phosphate buffer (5 mM, pH7.5) at 50 °C up to 72 h.

The reusability of selected samples in the catalyzing esterification reaction was evaluated. Immobilized lipase preparations were applied to catalyze the esterification reaction between oleic acid and butanol at 50 °C for 2 h. After each cycle, the biocatalyst was recovered via vacuum filtration and washed with heptane three times, after which the recovered biocatalyst was vacuum-dried at 35 °C for 1.5 h and was then reused in another cycle. The esterification capacity for each cycle was determined and compared with its initial esterification capacity that was considered to be 100%.

### 3.8. Statistical Analysis

Without specification in the above methods, all experiments were performed in triplicates. A one-way analysis of variance (ANOVA) was applied to evaluate the significance of differences among mean values at a confidence level of 95%. Tukey’s multiple comparison test was used to compares the significance of differences between each pair of means (*p* ≤ 0.05).

## 4. Conclusions

This work demonstrated, for the first time, the effectiveness of PEI or CS with a MW ≤ 2 kDa as a bridging unit to in situ crosslink the lipase physically adsorbed on a hydrophobic carrier, thus improving its stability. PEI and CS with a small MW showed strong diffusional capacity, as well as less risks in covering the active center of the lipase, with steric hindrance for substrates. These crosslinking strategies appear to be powerful and easy-to-perform for industrial applications because of their simplicity and time-saving nature, especially for the ones modified with CS, which is an inexpensive and renewable cationic polymer. In addition, IL modified with CS exhibited improved affinity toward substrates with a short chain length, indicating a high potential in modulating the catalytic properties of TLL for a specific reaction.

## Figures and Tables

**Table 1:** Factors and responses in factor screening experimental design and Gibbs free energy for each run.

Run	Factor A: LIPASE Concentration (mg/mL)	Factor B: Ethanol (%, *v*/*v*)	Factor C: NaCl (mol/L)	Factor D: Temperature (°C)	Factor E: pH	Response 1: Protein Loading Amount (mg/g Wet Support)	Response 2: Activity (U/g Wet Support)	Response 3: Specific Activity (U/mg Protein)	Immobilization Yield of Protein (%)	Immobilization Yield of Activity (%)	ΔG (kJ/mol)
1	2.00	30.00	0.00	25	3	11.99	0.10	0.01	19.98	0.00	−0.32
2	2.00	21.00	0.46	10	5	45.58	7939.23	174.20	75.96	32.26	−2.54
3	0.10	21.00	1.00	40	8	3.03	297.20	97.95	101.14	18.14	−4.78
4	0.10	0.00	1.00	25	8	3.02	886.66	244.96	100.65	45.36	−5.42
5	2.00	21.00	0.46	10	5	42.71	12,542.20	293.69	71.18	54.39	−2.73
6	0.10	0.00	0.39	40	3	2.41	360.78	149.66	80.35	27.72	−7.09
7	2.00	30.00	0.00	25	3	12.24	0.10	0.01	20.40	0.00	−0.37
8	1.04	18.45	1.00	10	3	5.66	185.85	32.83	18.10	6.08	−0.77
9	1.05	12.00	0.00	40	5	27.89	11,969.30	429.09	88.55	79.46	−4.07
10	2.00	0.00	1.00	25	5	54.30	9789.33	180.29	90.50	33.39	−3.67
11	0.10	30.00	0.00	25	8	1.45	107.32	73.79	48.48	13.66	−1.57
12	2.00	0.00	1.00	25	5	48.18	10,956.10	227.40	80.30	42.11	−3.43
13	2.00	0.00	0.00	10	8	50.31	2970.71	59.05	83.85	10.94	−3.28
14	1.04	18.45	1.00	10	3	7.77	1.96	0.25	24.83	0.05	−0.48
15	2.00	0.00	0.00	10	8	48.35	3329.86	68.87	80.58	12.75	−3.35
16	0.59	0.00	0.00	25	8	18.30	4298.99	234.94	102.68	43.51	−5.37
17	1.92	4.50	0.50	40	3	27.99	0.10	0.00	48.49	0.00	−1.47
18	0.10	30.00	0.60	10	5	2.25	248.77	110.72	74.90	20.50	−2.69
19	0.94	30.00	0.41	40	3	0.90	0.10	0.11	3.20	0.02	0.06
20	2.00	30.00	1.00	40	8	14.51	0.10	0.01	24.18	0.00	−0.53

**Table 2:** Summary on modification conditions.

Modification	Sample Code	Additives		GA Concentration (%, *v*/*v*)	Time
		Molecular Weight (kDa)/Viscosity (cp)	Concentration (%, *w*/*w*)		
Physical coating	PEI-L-1	2 kDa	30	0	60 min
	PEI-L-2		0.5		60 min
	PEI-L-3		0.5		24 h
	PEI-M-1	25 kDa	30		60 min
	PEI-M-2		0.5		60 min
	PEI-M-3		0.5		24 h
	PEI-H-1	75 kDa	30		60 min
	PEI-H-2		0.5		60 min
	PEI-H-3		0.5		24 h
	CS-L	≤2 kDa	0.5		60 min
	CS-M	50–190 kDa			
	CS-H	200–800 cp			
Chemical crosslinking	GA	/	/	1	15 min
	G-PEI-L-1	2 kDa	0.5		15 min
	G-PEI-M-1	25 kDa			
	G-PEI-H-1	75 kDa			
	G-PEI-L-2	2 kDa	0.5		PEI coating for 45 min + GA crosslinking for 15 min
	G-PEI-M-2	25 kDa			
	G-PEI-H-2	75 kDa			
	G-CS-L-1	≤2 kDa	0.5		15 min
	G-CS-M-1	50–190 kDa			
	G-CS-H-1	200–800 cp			
	G-CS-L-2	≤2 kDa	0.5		CS coating for 45 min + GA crosslinking for 15 min
	G-CS-M-2	50–190 kDa			
	G-CS-H-2	200–800 cp			

Note: L, M and H means low, medium, and high molecular mass, respectively.

## Data Availability

Not applicable.

## Supplementary Materials

Supplementary materials can be found at: https://www.mdpi.com/article/10.3390/ijms23062917/s1.
