# Supplementary material for: Characteristics of Crosslinking Polymers Play Major Roles in Improving the Stability and Catalytic Properties of Immobilized Thermomyces lanuginosus Lipase
Source: Int J Mol Sci. 2022 Mar 8;23(6):2917. doi: 10.3390/ijms23062917 (PMC8953303; doi:10.3390/ijms23062917)
Supplement: Supplementary file 1 [file ijms-23-02917-s001.zip › ijms-1608436-supplementary.pdf]

Article

Characteristics of Crosslinking Polymers Play Major Roles in Improving the Stability and Catalytic Properties of Immobilized *Thermomyces lanuginosus* Lipase

Yuhong Mao [1,*], Zhenling Cai [1], Chenxi Zhou [1], Hangzhen Lan [2] and Xiuyun Ye [1]

[1] Fujian Key Laboratory of Marine Enzyme Engineering, College of Biological Science and Technology, Fuzhou University, Fuzhou 350116, China; caizhenling0824@163.com (Z.C.); zcx5621175@163.com (C.Z.); xiuyunye@fzu.edu.cn (X.Y.)

[2] State Key Laboratory for Managing Biotic and Chemical Threats to the Quality and Safety of Agro-Products, College of Food and Pharmaceutical Sciences, Ningbo University, Ningbo 315211, China; hangzhen.lan@nbu.edu.cn

* Correspondence: yuhong.mao@fzu.edu.cn

Citation: Mao, Y.; Cai, Z.; Zhou, C.; Lan, H.; Ye, X. Characteristics of Crosslinking Polymers Play Major Roles in Improving the Stability and Catalytic Properties of Immobilized *Thermomyces lanuginosus* Lipase. *Int. J. Mol. Sci.* **2022**, *23*, 2917. https://doi.org/10.3390/ijms23062917

Academic Editor: Jesús Fernandez Lucas

Received: 7 February 2022
Accepted: 4 March 2022
Published: 8 March 2022

Publisher's Note: MDPI stays neutral with regard to jurisdictional claims in published maps and institutional affiliations.

Copyright: © 2022 by the authors. Submitted for possible open access publication under the terms and conditions of the Creative Commons Attribution (CC BY) license (https://creativecommons.org/licenses/by/4.0/).

Table S1. Significance analysis for responses yield, activity and specific activity.

	Significance (P values)					
	Yield		Activity		Specific activity	
Model		< 0.0001		< 0.0001		< 0.0001
	A	< 0.0001	A	0.0055	E	< 0.0001
	B	< 0.0001	B	< 0.0001	DE	0.0061
Factors	E	< 0.0001	C	0.0008		
	AB	0.0023	E	< 0.0001		
	AD	0.0006	AE	0.0068		
Lack of Fit		0.5743		0.9258		0.9281

Note: factor A-lipase concentration (mg/mL); factor B-ethonal fraction (%, v/v); factor C-NaCl addition (mol/L); factor D-temperature (°C); factor E-pH. .

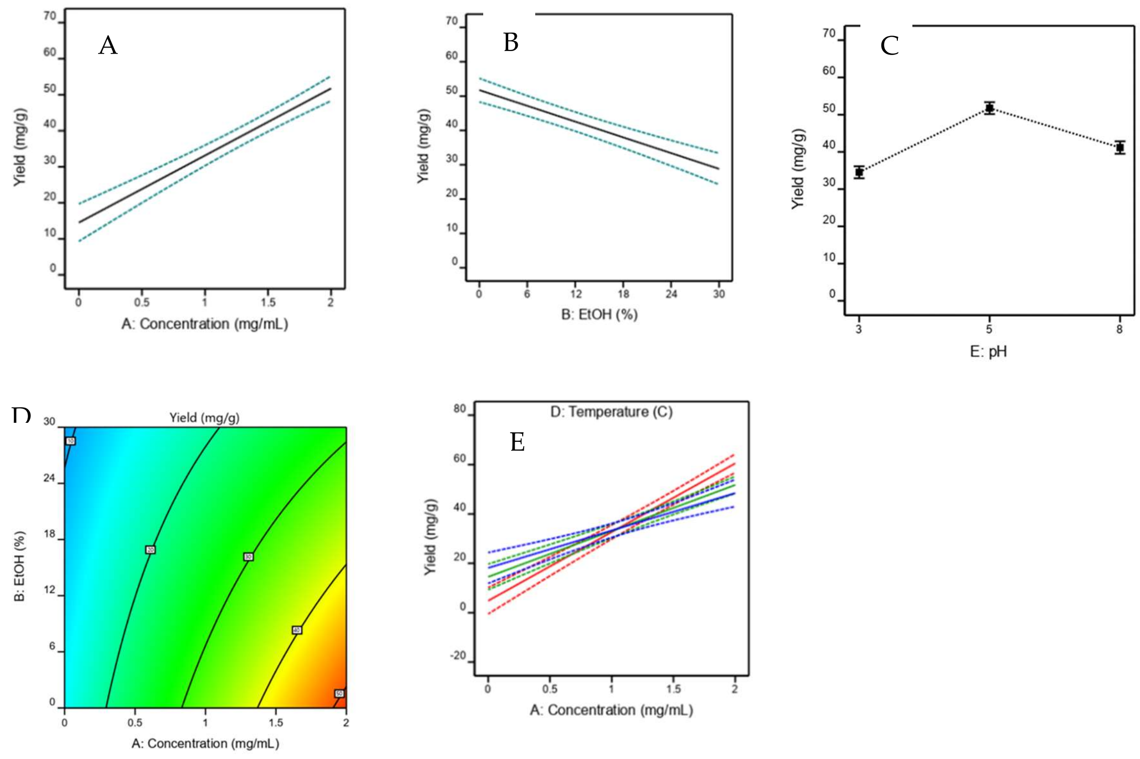

Figure S1. The influence of significant factors on the immobilization yield. (A) concentration; (B) ethanol fraction; (C) pH values; (D) interaction between ethanol fraction and concentration; (E) interaction between temperature and concentration.

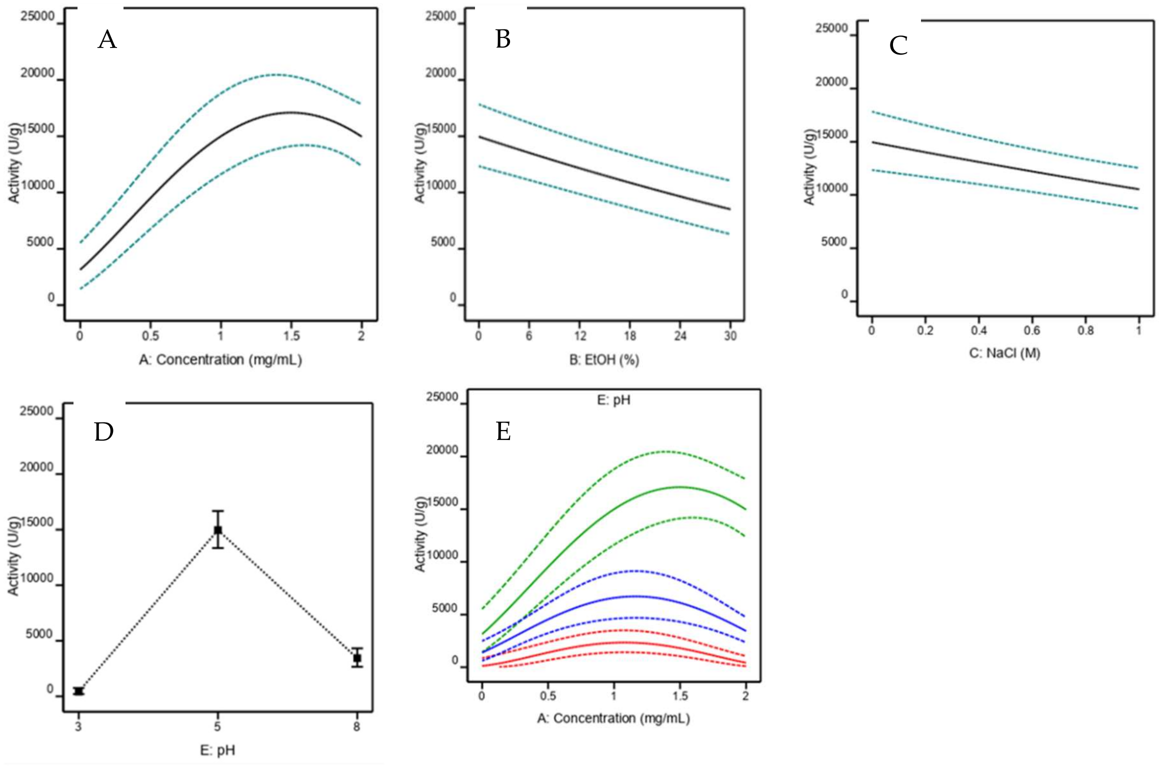

Figure S2. The influence of significant factors on the activity. (A) concentration; (B) ethanol fraction; (C) NaCl concentration; (D) pH values; (E) interaction between pH values and concentration.

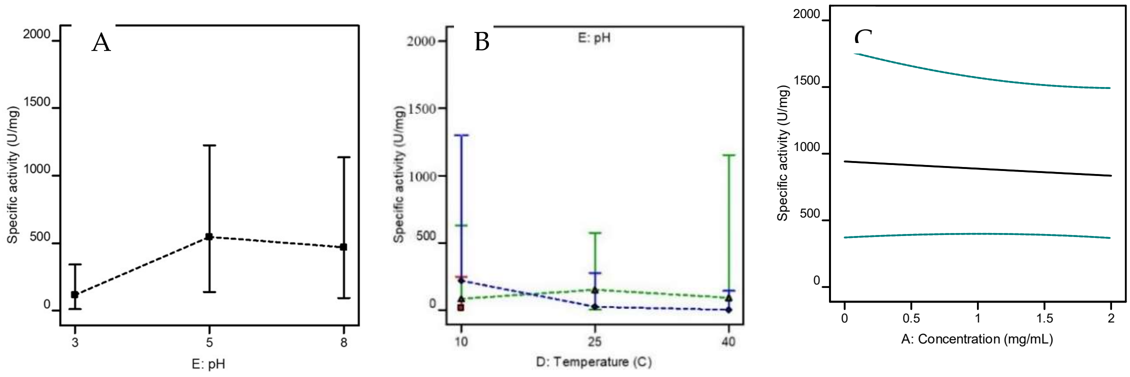

Figure S3. The influence of significant factors on the specific activity. (A) pH values; (B) interaction between pH and temperature; (C) concentration (insignificant).

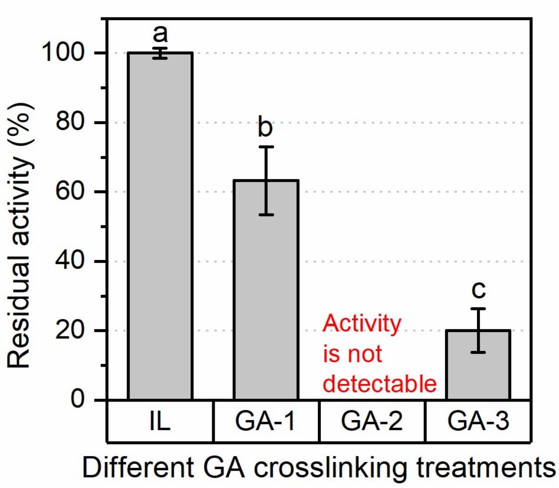

Figure S4. Influence of different glutaraldehyde (GA) treatments on the residual activity. IL: immobilized lipase via adsorption; GA-1: IL crosslinked via 1% GA for 15 min; GA-2: IL crosslinked via 10% GA for 15 min; GA-3: IL crosslinked via 1% GA for 1 h.

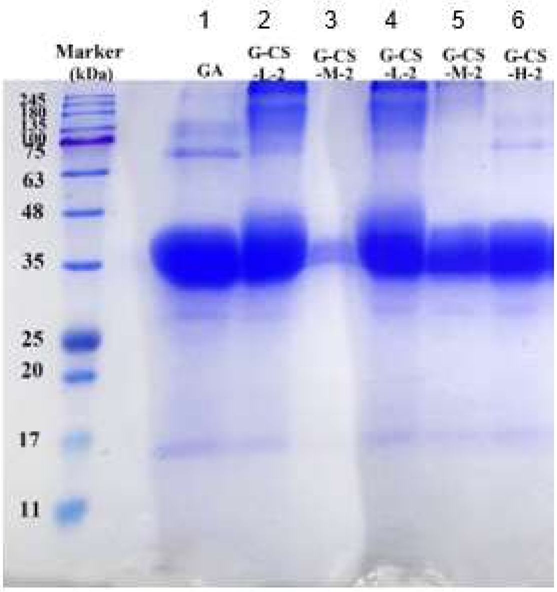

Figure S5. Original SDS-PAGE pattern of various modified immobilized lipase preparations. Lane 3 was due to an experimental mistake.
